# Multi-omics insights into plant-microbe dysbiosis caused by cyanobacterial bloom-affected water

**DOI:** 10.1016/j.crmicr.2025.100500

**Published:** 2025-10-23

**Authors:** Minsoo Jeong, Soeun Park, Seungjin Jeong, Surye Park, Sohyun Yeo, Bomi Ryu, Jae-Ho Shin, Seungjun Lee

**Affiliations:** a Department of Applied Biosciences, Kyungpook National University, Daegu, 41566, Republic of Korea; b Department of Food Science and Nutrition, Pukyong National University, Busan, 48513, Republic of Korea; c Department of Integrative Biotechnology, Kyungpook National University, Daegu, 41566, Republic of Korea; d NGS Core Facility, Kyungpook National University, Daegu, 41566, Republic of Korea

**Keywords:** Cyanotoxins, Lettuce, Metabolome, Hydroponic, Nakdong River, Phytotoxicity

## Abstract

The use of untreated, cyanobacterial bloom–affected river water as a readily available source for crop production is a realistic scenario in many regions, yet its systemic impact on crop health remains poorly defined. Here, we investigate the multifaceted effects of this practice by cultivating lettuce (*Lactuca sativa*) with bloom-season water from the eutrophic Nakdong River and comparing it to lettuce grown in a nutrient-optimized hydroponic solution. We found that exposure to bloom-affected water severely inhibited plant growth and led to the accumulation of microcystins in edible tissues, with estimated daily intakes exceeding WHO safety thresholds. Multi-omics analyses revealed that this phenotype was driven by a synergistic failure of internal and external support systems: key mitochondrial genes for energy production were downregulated, protective antioxidant flavonoids were depleted, and the aquatic microbiome shifted to a dysbiotic state that favored stress-tolerant taxa while reducing beneficial ones. Together, these results establish how bloom-affected water initiates a vicious cycle of physiological stress and microbial dysbiosis that undermines crop health. This study provides an integrative framework for assessing risks in real-world hydroponic systems and for guiding future investigations into more complex soil-based agriculture.

## Highlights

• Toxic cyanobacteria-contaminated irrigation water inhibited lettuce growth and development.
• Genes related to energy metabolism were downregulated in response to cyanobacteria exposure.
• Anti-inflammatory and antioxidant metabolite levels decreased in cyanobacteria-treated lettuce.
• Pathogenic Pseudomonas species increased 2.3-fold in lettuce exposed to toxic cyanobacteria

## Graphical abstract

**Figure:**
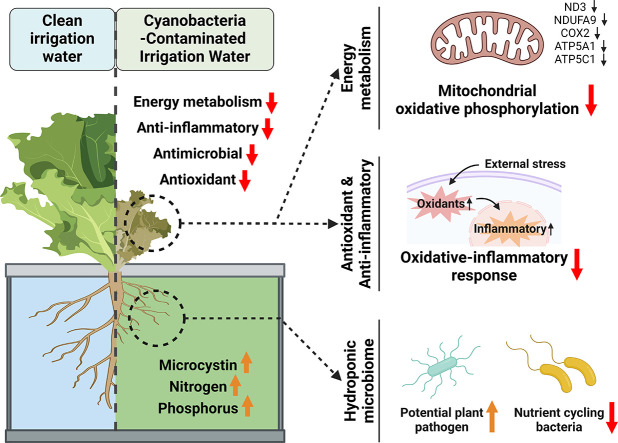

## 1 Introduction

Agricultural water affected by harmful cyanobacterial blooms, which produce toxic compounds collectively referred to as cyanotoxins, threatens global food security (Ji and Lee, 2022; Wang et al., 2022). During the cyanobacterial bloom season, which typically spans from late spring to early fall, previous studies have demonstrated that cyanotoxins can be absorbed and translocated within plant tissues, thereby posing potential risks to both crop development and human health (du Plessis, 2022; Moreira et al., 2022). In major rivers and lakes around the world, recurrent cyanobacterial blooms have emerged as a significant threat to regional food safety and public health, particularly because these eutrophic water bodies are commonly used as water sources in both soil-based and hydroponic farming systems (Kim et al., 2022; Park et al., 2021).

The crisis of water contamination with toxic cyanobacteria reflects a global trend exacerbated by climate change and anthropogenic eutrophication (Reinl et al., 2023). During the bloom season, cyanotoxins such as microcystins are among the most commonly detected in freshwater environments, with the specific profile and concentration varying depending on the bloom's species composition. For example, the Nakdong River in South Korea has reported concentrations of microcystins reaching up to 5,000.0 μg L⁻¹, far exceeding the World Health Organization’s drinking water guideline of 1.0 μg L⁻¹ (Kim et al., 2022; Park et al., 2021). The blooms impact agricultural systems beyond the aquatic environment, as use of bloom-affected water transfers various compounds including cyanotoxins to crops, posing health risks to humans and animals (Kruk et al., 2023). Ultimately, this contamination creates pathways for cyanotoxins to enter the food chain and disrupts both soil and aquatic ecosystems, underscoring the need for holistic environmental strategies (Hernández-García and Martínez-Jerónimo, 2023; Zepernick et al., 2023).

Despite the alarming levels of contamination with cyanotoxins in freshwater, field-relevant evidence demonstrating the physiological, molecular, and ecological effects of such exposure on crop systems remains scarce. Previous studies have reported microcystin accumulation in agricultural crops, with toxin concentrations detected in lettuce (*Lactuca sativa*) leaves from 33.0 to 143.4 μg kg⁻¹ (dry weight) and in carrot roots up to 5.2 μg g⁻¹(dry weight) (Bittencourt-Oliveira et al., 2014; Machado et al., 2017). These findings, though alarming, only scratch the surface of a complex threat to food safety. It is important to note, however, that the role of cyanobacteria is not exclusively detrimental. Certain species are known to provide benefits to agricultural systems, such as enhancing soil fertility through nitrogen fixation, and are even utilized as bio-fertilizers (Massa et al., 2024; Santos et al., 2022). This highlights the complex nature of bloom-affected water, which contains a mixture of potentially harmful compounds alongside beneficial microbes and nutrients. Therefore, understanding the net outcome of using such water is essential for a comprehensive risk assessment. Current understanding of microcystin effects on plant physiology remains limited to observable outcomes, such as reduced growth, altered photosynthesis, and visible stress symptoms (Hernández-García and Martínez-Jerónimo, 2023).

However, the molecular responses underlying these changes, and their interactions with plant-associated microbiota, are largely unexplored. This knowledge gap restricts the development of holistic mitigation strategies. Particularly, field-relevant experiments using actual bloom-affected water remain scarce. This is critical because, unlike studies using purified toxins, real-world water sources harbor diverse chemical compounds and complex microbes that can influence the uptake and biotransformation of bloom-derived compounds. When water containing these complex mixtures is applied to crops, the dynamics of plant-microbe associations may be altered, with potential consequences for plant physiology, resilience, and nutrient cycling.

To address this knowledge gap, this study simulates a highly relevant agricultural scenario: the use of untreated water from the Nakdong River, a complex matrix composed of dissolved nutrients, cyanotoxins, and a diverse array of microbial taxa, for hydroponic production, a common practice in certain regions, including South Korea, due to economic or infrastructural constraints (Jung et al., 2016). Recognizing that the effects of bloom-affected water in this context are likely complex and multifactorial, we employed an integrated multi-omics approach. This approach allows for a more holistic, systems-level view by seeking to: 1) link observable physiological stress, 2) underlying alterations in gene expression and metabolic networks, and 3) investigate how these internal plant responses are associated with shifts in the external aquatic microbiome.

By integrating these approaches, this research seeks a holistic understanding of how bloom-affected water impacts crop systems. This work provides a critical foundation for assessing the risks in real-world hydroponic systems and for future investigations into more complex soil-based agriculture, ultimately advancing our knowledge of plant-toxin-microbe interactions and informing the development of more resilient agricultural practices.

## 2 Materials and methods

### 2.1 Water sampling and quality analysis

#### 2.1.1 Collection of water samples

Water samples for a hydroponic system were collected in August 2022 from the Nakdong River at Daedong Wharf, Gimhae, Gyeongsangnam-do, South Korea (GPS coordinates: 35.2403°N, 128.9975°E) during a season of cyanobacterial bloom. This site is representative of water sources commonly used by local farmers for agricultural cultivation, including both soil-based farming and hydroponic systems, highlighting the potential risks associated with using bloom-affected river water in agriculture. The samples were collected at a depth of 0.5 m below the surface from three distinct points within a 10-meter radius to ensure spatial representativeness, following standard freshwater sampling procedures stipulated by the Ministry of Environment, Republic of Korea (Standard Methods for Water Quality, ES 04351.1b). This protocol includes collecting sub-surface water (approx. 0.5 m depth) in sterile containers and transporting them under refrigerated conditions (4 °C) to maintain sample integrity.. A volume of 2.5 L was used for each hydroponic system. The collected samples were transported to the laboratory in sterile containers maintained at 4 °C to preserve sample integrity. Upon arrival, the water was immediately stored at 4 °C in the dark in sealed, sterile polypropylene containers (5 L capacity). All water used throughout the 30-day experiment was drawn from this single-batch storage. Maximum storage duration was 35 days (August 15 - September 19, 2022). This single-batch approach eliminated day-to-day variability in bloom composition and ensured experimental reproducibility. As detailed in the Results, the bloom was primarily composed of cyanobacteria from the genera Microcystis and Dolichospermum. This composition is consistent with typical harmful algal blooms observed during the summer season in this river.

#### 2.1.2 Measurement of water quality parameters

Water quality analysis included measurements of total nitrogen (N), total phosphorus (P), cyanobacterial counts, and microcystin levels. To assess the degree of eutrophication, total N and P concentrations were measured using a DR/1900 Portable Spectrophotometer (Hach Company, Loveland, Colorado, USA) according to the manufacturer’s instructions. Cyanobacterial counts were determined using the Water Environment Information System (http://211.114.21.27/web). Microcystin concentrations were measured using the Microcystins-ADDA ELISA plate kit (Eurofins Abraxis, Warminster, Pennsylvania, USA), with a detection range of 0.15 to 5.00 μg L⁻¹. All standards, controls, and samples were analyzed in triplicate.

### 2.2 Crop cultivation system

#### 2.2.1 Experimental design

Green lettuce (*Lactuca sativa* var. *crispa* L.) seeds were germinated using sterilized cotton with 150 mL of deionized water for 10 days. After germination, seedlings were transferred to a hydroponic system set up in a plant-growing chamber (SPZW-A01WU1, Jiangsu Skyplant Greenhouse Technology Co., China). The system consisted of plastic trays with a water capacity of 2.5 L, equipped with an air pump for aeration and a perforated polystyrene board to support the plants, following standard hydroponic configurations (Park et al., 2021). The chamber was maintained at 20–22 °C with a 12 h light (approximately 250 μmol m⁻² s⁻¹) and 12 h dark cycle. Air in the ventilation system was filtered using 110 mm qualitative filter papers (Cat No. F1093-110, Chmlab Group, Terrassa-Barcelona, Spain).

The experimental design intentionally compared two realistic agricultural scenarios: optimal hydroponic conditions (control group with balanced nutrients) versus bloom-affected Nakdong River water conditions (NRW group). This approach, while creating inherent differences in nutrient profiles, was chosen to reflect the practical trade-offs faced in agricultural water management. This is particularly relevant given that hydroponic systems using untreated natural water sources are increasingly common in cost-sensitive agricultural operations across Asia, where advanced water treatment infrastructure may not be readily available or economically feasible. This design, therefore, provides ecologically relevant insights into the net impact of using such water sources.

Two groups were established: the deionized water (Control) group, where a nutrient solution was prepared using a commercial hydroponic fertilizer (A and B, Daeyu Co., South Korea) formulated based on the standard Hoagland solution. The detailed elemental composition of the stock solutions is provided in Table S1. The solution was diluted to achieve final concentrations consistent with optimal lettuce nutrient requirements. The NRW group used Nakdong River water containing cyanobacterial blooms without additional nutrients. Each group had three biological replicates, with each replicate containing five lettuce plants (n=15 plants per treatment). Water levels in the hydroponic systems were monitored daily, and when the water volume decreased by 500 mL from the initial 2.5 L, it was replenished with 500 mL of the respective treatment solution—Nakdong River water for the NRW group or nutrient solution for the control group—to maintain consistent nutrient concentrations and pH levels throughout the cultivation period. Water replenishment was necessary throughout the experiment to compensate for evapotranspiration. To minimize temporal variability, all NRW was collected from a single sampling event, homogenized, and stored under controlled conditions, thereby eliminating the substantial day-to-day fluctuations in bloom composition that would occur with fresh river water collection. This study was intentionally designed with asymmetrical timescales to distinguish between the ecological process of microbial community assembly and the cumulative, agriculturally relevant outcome in the host plant. Accordingly, we employed a short-term, time-series analysis (days 0-5) to characterize the dynamic succession of the aquatic microbiome, and a long-term, end-point analysis (day 30) to assess the final, integrated impact on lettuce growth and its molecular state at harvest. The experimental sampling was conducted on two distinct timescales. For microbial community analysis, water samples from the NRW group were collected daily from Day 0 to Day 5 (n=3 biological replicates per time point), whereas the control group was sampled only at Day 0 (n=3 biological replicates) to serve as a stable baseline. For all other analyses—plant growth metrics, transcriptomics, metabolomics, and microcystin quantification—lettuce samples were harvested at the end-point of Day 30 (n=3 biological replicates per group, with growth metrics measured on all 15 plants per group).

### 2.3 Measuring parameters of lettuce growth and development

#### 2.3.1 Leaf and root length and weight

Lettuce samples were harvested after 30 days of hydroponic cultivation, marking the end of the growth period under controlled conditions. After harvesting the lettuce samples, the lengths and weights of the lettuce leaves and roots were measured. The measurements included the length of the longest leaf and root, as well as the fresh weights of the leaves and roots. Measurements were conducted across all three biological replicates per group, with each replicate consisting of five plants (n=15 plants per group), using a digital caliper (Mitutoyo, Japan) for length and an analytical balance (Sartorius, Germany) for weight, with data recorded as means ± standard deviations to ensure statistical reliability.

### 2.4 Microcystin extraction and quantification

#### 2.4.1 Extraction procedure for lettuce samples

Microcystins were extracted from harvested lettuce leaves using a previously established method (Chen et al., 2010; Gutiérrez-Praena et al., 2014). Ten grams of lettuce leaves were ground with 50.00 mL of 50 % methanol. The ground samples were sonicated for 30 minutes and then centrifuged at 1,800 × g for 5 minutes at 4 °C. The supernatant was filtered using a Buchner funnel with qualitative analysis filter paper (110 mm, Cat No. F1093-110, Chmlab Group, Terrassa-Barcelona, Spain). The filtered sample supernatant was concentrated in a water bath at 60 °C using a rotary evaporator (Buchi® Rotavapor® R-215, Buchi, Meierseggstrasse, Flawil, Switzerland) until approximately 1 mL of supernatant remained. The concentrated supernatant was rinsed with about 3.00 mL of 50 % methanol and subjected to solid phase extraction using an Oasis HLB 6 cc Vac Cartridge (500 mg Sorbent per Cartridge, 60 μm, Waters, Milford, Massachusetts, USA). The cartridges were washed with 20.00 mL of 100 % methanol and 20 mL of deionized water, followed by rinsing with 20 mL of 10 % methanol. The final eluates were obtained using 20 mL of 80 % methanol, concentrated with nitrogen at 80 °C using an MD200 Series Sample Concentrator (Allsheng, Hangzhou, China), and dissolved in 1 mL deionized water. To validate the extraction method and account for matrix effects, recovery efficiencies for microcystin congeners (MC-LR, MC-RR, MC-YR) were determined by spiking lettuce samples with known concentrations (10 μg kg^-1^) of each standard, yielding recoveries of 85-92 % for MC-LR, 80-88 % for MC-RR, and 82-90 % for MC-YR, consistent with established protocols (Chen et al., 2010). For LC-MS/MS analysis, the eluate was filtered using plastic two-piece syringes (2 mL, S7515-3, Thermo Fisher Scientific, Waltham, Massachusetts, USA) and 0.13 cm PVDF membranes (Syringe Driven Filters, 0.22 μm, non-sterile, DNase/RNase free, non-pyrogenic, FPV-223-013, Jet Biofil, Guangzhou, China).

#### 2.4.2 Analytical conditions for LC-MS/MS

The concentrations of three microcystin congeners, specifically microcystin-LR (leucine-arginine), microcystin-RR (arginine-arginine), and microcystin-YR (tyrosine-arginine), were measured in the prepared lettuce leaf extracts. These variants were selected as they are consistently reported as the most dominant and abundant in the Nakdong River during bloom events (Kim et al., 2022; Park et al., 2021). The LC system consisted of an ACQUITY I CLASS PLUS, and the MS system consisted of a Xevo TQ-S micro (WATERS, Milford, Massachusetts, USA). Chromatographic separation was conducted on a BEH C18 column (2.1 mm × 100 mm, 1.7 μm, WATERS, Milford, Massachusetts, USA) at 35 °C with a flow rate of 0.30 mL/min. The solvents used were 0.1 % formic acid (solvent A) and 0.1 % formic acid acetonitrile (solvent B), with a binary gradient of 95 % A/5 % B to 100 % B over 5.50-7.50 minutes, followed by re-equilibration to 95 % A/5 % B. Mass spectrometric analysis was performed using an electrospray ionization source operating in the positive ion mode. The instrument parameters were set as follows: capillary voltage of 3.00 kV, cone voltage of 60 V, and desolvation temperature of 500 °C. To ensure analytical accuracy and account for matrix effects in lettuce tissues, the LC-MS/MS method was validated using spiked samples with known concentrations (10.00 μg kg^-1^) of MC-LR, MC-RR, and MC-YR standards, achieving limits of detection (LOD) of 0.05 μg kg^-1^ and limits of quantification (LOQ) of 0.15 μg kg^-1^, with calibration curves showing linearity (R² > 0.99) across a range of 0.1-50 μg kg^-1^ (Chen et al., 2010).

#### 2.4.3 Calculation of estimated daily intake

The estimated daily intake (EDI) of microcystins from lettuce consumption was calculated based on the WHO’s tolerable daily intake (TDI) guideline of 0.04 μg kg^-1^ body weight/day for microcystin-LR (WHO, 2020). To provide a conservative, health-protective assessment, the total microcystin exposure was estimated by summing the concentrations of all detected variants (MC-LR and MC-RR). While acknowledging that other congeners like MC-RR exhibit lower toxicity than MC-LR, this approach is often used for a preliminary risk assessment in the absence of universally accepted toxic equivalency factors (TEFs) for all dietary exposure scenarios. The mean weight of Korean adults in 2021 was 74.90 kg for men and 58.89 kg for women (Korea Statistical Information Service, https://kosis.kr/index/index.do). The total daily intake of vegetables by adults was approximately 274.00 g (National Nutrition Statistics, Korea Health Industry Development Institute, https://www.khidi.or.kr/nutristat). EDI was computed by multiplying the measured concentrations of microcystin variants (MC-LR, MC-RR, MC-YR) in lettuce (μg kg^-1^ dry wt.) by the daily vegetable intake (274.00 g, converted to dry wt. assuming 90 % water content, i.e., 27.40 g dry wt.), divided by the mean body weight (kg), and expressed as μg kg^-1^ body weight/day; calculations included all detected variants (MC-LR, MC-RR, MC-YR) to assess total exposure, with results compared against the WHO TDI for MC-LR as the reference standard.

$$\mathrm{EDI}=\;\frac{Concentration\;of\;microcystins\;\left(\mu g/kg\right)\;\times Daily\;consumption\left(kg\right)}{Body\;weight\left(kg\right)}$$

#### 2.4.4 Calculation of trophic state index

To assess the trophic state of the Nakdong River water samples, the Trophic State Index (TSI) was calculated based on both total phosphorus (TP) and total nitrogen (TN) concentrations. The TSI is a widely used metric for quantifying the nutrient status of water bodies and predicting the potential for eutrophication. The TSI for TP was calculated using the Carlson Trophic State Index (CTSI) formula, as described by Carlson (1977), where TP is expressed in μg L^-1^; this index was originally developed using Transparency (SD), Chlorophyll-a (Chl-a), and TP, though only TP was applied here due to data availability. The TSI for TN was calculated using the Kratzer and Brezonik formula, where TN is expressed in mg L^-1^, adapted for nitrogen-based eutrophication assessment (Kratzer and Brezonik, 1981). The specific formulas are presented in the accompanying equations. The formulas for TSI-TP and TSI-TN are as follows:

TSI(TP)=14.42×In(TP)+4.15

TSI(TN)=14.43×In(TN)+54.45

### 2.5 Analysis of microbial communities

#### 2.5.1 DNA extraction procedure from water samples

One hundred milliliters of each collected water samples from the hydroponic system were filtered through a sterile 0.22 μm membrane filter (47 mm, Whatman, UK) using an Alligator 200 Diaphragm Liquid Pump (Rocker, Taiwan) and a MultiVac 601 – MB – A Multi-Branch Filtration System (Rocker, Taiwan). Three replicate water samples were collected per treatment group (NRW and Control) to ensure robustness in microbial community profiling. The control group was sampled at Day 0, representing the baseline stable condition, while the NRW group was sampled daily from Day 0 to Day 5 to capture the expected dynamic changes. While the root-associated microbiome is undoubtedly critical for plant health, this study's primary objective was to characterize the microbial community dynamics within the hydroponic water itself, as it represents the direct source of microbial and chemical stressors. The DNA was extracted from the filters using the DNeasy® PowerSoil® Pro Kit (Cat. No. 47016, Qiagen, Hilden, Germany) according to the manufacturer’s protocol. DNA extraction was performed in triplicate for each filter to minimize technical variability, and extracted DNA quality was verified using a NanoDrop One Microvolume UV-Vis Spectrophotometer (Thermo Fisher Scientific, USA) to ensure A260/A280 ratios between 1.8 and 2.0.

#### 2.5.2 rRNA sequencing and bioinformatics workflow

For sequencing library preparation, the V4 region of the 16S rRNA gene was amplified using adapter TruSeq (Part# 15044223, Illumina, San Diego, CA, USA) with primers XT-V4-F (ACA CTC TTT CCC TAC ACG ACG CTC TTC CGA TCT GTG NCA GCM GCC GCG GRA) and XT-V4-R (GTG ACT GGA GTT CAG ACG TGT GCT CTT CCG ATC GAC TAC NVG GGT WTC TAA TC), targeting bacterial community. Sequencing was run for 300 cycles using the Miseq Reagent Kit v2, following the manufacturer’s protocol (Document Number. 15027617, Illumina, San Diego, CA, USA). Each library was prepared from three biological replicates per treatment group (NRW and Control), with each replicate sequenced to an average depth of 43,285 reads per sample (range: 17,483–94,571 reads), ensuring sufficient coverage for microbial diversity analysis. The libraries were sequenced at the next-generation sequencing core facility of Kyungpook National University (KNU NGS Core Facility, Daegu, South Korea) using the Illumina MiSeq platform system. The raw sequence data for bacterial communities were analyzed using the Quantitative Insights Into Microbial Ecology 2 (QIIME2, v. 2023.5) software package. DADA2 software was used to remove chimeric sequences and generate amplicon sequence variants (ASVs) (Callahan et al., 2016). Taxonomic assignments for the bacterial community were performed using Silva 138 SSURef NR99 515F/806R region sequences and corresponding taxonomy. R software version 4.3.1 and RStudio (ver. 2023.6.1.524) were used to analyze the microbiota of bacteria and fungi, including alpha diversity (Chao1, Simpson, and Shannon indices) and beta diversity (Bray-Curtis method) at the ASVs level. The similarity and abundance of microbes between experimental groups were visualized using heatmaps, and overlapping microbial differences were displayed.

### 2.6 Transcriptomic analysis

#### 2.6.1 RNA extraction and quality control

To identify differences in gene expression between NRW and Control groups, RNA was extracted from the lettuce leaves. The extraction was performed using the RNeasy Plant Mini Kit (Cat. No. 74904, Qiagen, Hilden, Germany) following the manufacturer's protocol. RNA was also extracted from three biological replicates per treatment group (NRW and Control), with each replicate consisting of leaves from five plants (n=15 plants per group). The quality of the extracted RNA was assessed using a Nanodrop spectrophotometer to ensure A260/A280 ratios between 1.8 and 2.2 and A260/A230 ratios greater than 2.0. Additionally, RNA integrity was checked using the 4150 TapeStation System (Agilent, MA, USA), with the RNA integrity number used to confirm RNA quality. Extracted RNA from each replicate was kept separate during quality assessment, and only samples with an RNA Integrity Number (RIN) greater than 7 were used; subsequently, RNA from the three replicates per group was pooled for each treatment group to reduce technical variability in downstream sequencing. The concentration of purified mRNA was verified using the 4150 TapeStation System (Agilent, MA, USA).

#### 2.6.2 RNA sequencing and data analysis

To enrich for mRNA transcripts, poly(A) RNA was isolated from the total RNA samples using the PolyATtract® mRNA Isolation System (Promega, Madison, WI, USA) according to the manufacturer's protocol, prior to library construction. For library preparation, the MGIEasy RNA Directional Library Prep Set (MGI Tech, Wuhan, China) was used. RNA sequencing was performed on the DNBSEQ-G400RS (PE100) platform (MGI Tech, Wuhan, China) at the Molecular Microbiology Laboratory, Department of Applied Life Sciences, Kyungpook National University (Daegu, South Korea). The paired-end reads generated from RNA-seq were processed through a bioinformatics pipeline to quantify gene expression. Initially, raw reads were aligned to the Ensembl reference genome using HISAT2 (Kim et al., 2019). Subsequently, the aligned reads were used to assemble transcripts and estimate their abundance using StringTie (Pertea et al., 2016), with expression levels quantified as Fragments Per Kilobase of transcript per Million mapped reads (FPKM). For differential expression analysis, the raw read counts for each gene were then analyzed in R software using the DESeq2 package (v1.30.1). Genes were considered to be differentially expressed if they met the statistical thresholds of a false discovery rate (FDR) adjusted p-value < 0.05 and an absolute log2(fold change) > 1.RNA sequencing was conducted on pooled samples from three biological replicates per treatment group (NRW and Control), as described in Section 2.5.1, with an average sequencing depth of 20 million reads per sample to ensure sufficient coverage for gene expression analysis. The RNA-seq expression values were measured in FPKM (fragments per kilobase of transcript per million mapped reads). Subsequent statistical analysis was performed in R software, using DESeq2 results to identify differentially expressed genes (Love et al., 2014)**.**

### 2.7 Phytochemical profiling of lettuce

#### 2.7.1 Extraction of metabolites from lettuce leaves

Phytochemical analysis of lettuce (*Lactuca sativa* var. *crispa* L.) from the NRW and control groups was performed to compare the composition of phytochemicals (Yang et al., 2018). One gram of lettuce leaves was sonicated for 30 minutes with 10 mL of 80 % methanol containing 1 % formic acid. To ensure accurate extraction, 50 μg mL^-1^ of chlorogenic acid was added as an internal standard prior to sonication, allowing for validation of metabolite recovery and extraction efficiency (Park et al., 2021). After sonication, the sample was centrifuged at 4,000 RPM for 10 minutes. The supernatant was transferred to a new conical tube and stored at 4 °C overnight. After overnight storage, the sample was centrifuged again at 4,000 RPM for 10 minutes. The final supernatant was transferred to a 1.75 mL conical tube and centrifuged at 12,000 RPM for 10 minutes. The 120.00 μL of clear supernatant was transferred to a vial insert, and the sample was used for analysis (Qin et al., 2018).

#### 2.7.2 LC-Q-TOF MS/MS analysis conditions

Phytochemical analysis was conducted using a liquid chromatography-quadrupole time-of-flight (LC-Q-TOF) mass spectrometer. The analysis conditions included a flow rate of 0.3 mL min^-1^, a loading volume of 5.00 µL, and a column temperature of 40 °C using a Waters C18 column (2.1 × 100 mm, 1.7 µm particle size). The mobile phases consisted of solvent A, which was control with 0.1 % formic acid, and solvent B (Qin et al., 2018), which was acetonitrile with 0.1 % formic acid. The gradient elution program started with 100 % solvent A and 0 % solvent B, maintaining this composition for 1 minute. Over 25 minutes, the composition gradually shifted to 0 % solvent A and 100 % solvent B, which was held constant for 2 minutes, then rapidly returned to 100 % solvent A and 0 % solvent B and maintained for an additional 3 minutes. MS/MS analysis was performed using a SYNAPT XS high-resolution mass spectrometer (Waters, Milford, MA, USA) equipped with an electrospray ionization (ESI) source. The MS conditions were optimized to ensure accurate detection of phytochemicals. The mass spectrometer operated with a capillary voltage of 3 kV in positive mode and 2 kV in negative mode within the m/z range of 100–1300 Da. The sampling cone voltage was maintained at 40 V, and the source temperatures were set to 120 °C and 100 °C for positive and negative modes, respectively. The desolvation temperatures were 350 °C for positive mode and 250 °C for negative mode. The cone gas flow rate was maintained at 50 L/H, while the desolvation gas flow rate was 600 L/H. The MS Continuum mode utilized two functions, with low energy set at a trap collision energy of 6V and high energy employing a ramp trap collision energy of 20V to 45V. Specific phytochemicals were identified by comparing their accurate masses and MS/MS fragmentation patterns against the ChemSpider database and KEGG pathway annotations, and normalized to the internal standard (chlorogenic acid) added during extraction to ensure accurate metabolite concentrations.

#### 2.7.3 Metabolomic data processing and analysis

Data analysis was conducted using the Waters Progenesis QI software (Waters, Milford, MA, USA) which enabled us to generate a molecular formula with a mass accuracy limit of 5 ppm and an MS ≥ 95 (related to the contribution to mass accuracy, isotope abundance and isotope). The raw data obtained from the LC-Q-TOF analysis were processed in the software and then identified based on accurate mass and MS/MS fragments by searching ChemSpider ID and KEGG pathway, online public databases. The analysis identified a total of 11,873 features in positive mode and 17,484 features in negative mode. The phytochemical profile differences between NRW and control groups were analyzed, highlighting key features that showed significant changes in abundance between the two groups.

### 2.8 Statistical analysis

All statistical analyses were conducted using R software (ver 4.0.3). Prior to group comparisons, data were tested for normality using the Shapiro-Wilk test ("stats" package) and for homogeneity of variances using Levene's test ("car" package, ver 3.0-10). Based on the outcomes, statistical significance between the two independent groups was evaluated using one of the following: (i) the unpaired two-sample t-test when assumptions of both normality and equal variance were met, (ii) Welch's t-test when the normality assumption was met but the equal variance assumption was not, or (iii) the Wilcoxon rank-sum test when the assumption of normality was violated. To assess differences among three or more groups, a one-way analysis of variance (ANOVA) was performed, followed by Tukey’s post hoc test to determine specific group differences. Results were considered statistically significant at a probability value of *p* < 0.05.

## 3 Results

### 3.1 Characterization of toxin-producing cyanobacterial contamination and eutrophication in Nakdong River water

The experimental design compared nutrient-optimized hydroponic conditions (Control) with bloom-affected Nakdong River water conditions (NRW), representing the practical decision farmers face between controlled nutrient solutions and available natural water sources during bloom periods. Green lettuce (*Lactuca sativa* var. *crispa* L.) was used to assess the effects of cyanobacterial bloom-affected river water on plant growth. Water samples from the Nakdong River, known for its cyanobacterial blooms, revealed cyanobacterial cell counts of 144,062 cells/mL, representing the combined cells of harmful genera including *Microcystis, Dolichospermum* (formerly *Anabaena*), *Aphanizomenon*, and *Oscillatoria* (Table 1). Microcystin concentrations were approximately 1,447.0 μg L^-1^. Other cyanotoxins anatoxins, saxitoxins, and cylindrospermopsins were not detected (< 0.1 μg L^-1^). To evaluate the eutrophication status, total nitrogen (N) and total phosphorus (P) levels were analyzed, revealing nitrogen levels of 105.0 mg L^-1^ N and phosphorus levels of 26.9 mg L^-1^ PO₄³⁻. These values far exceed the eutrophication thresholds commonly observed in freshwater systems, indicating a highly eutrophic state and substantial pollution load. The TSI calculated from the total nitrogen concentration (TSI(TN)) was 147.77, while the TSI calculated from the total phosphorus concentration (TSI(TP)) was 221.1, both confirming a hyper-eutrophic state. The collected river water samples were thus deemed highly contaminated and suitable for use in hydroponic cultivation experiments to simulate real-world agricultural conditions.

**Table 1:** Water quality parameters of the collected water samples from Nakdong River sample.

		water sample
Microcystin concentrations (μg L^-1^)		1,447.0
Cyanobacterial counts (cells mL^-1^)		144,062.0
Total nitrogen (mg L^-1^ N)		105.0
Total phosphorus (mg L^-1^ P)	26.9	
TSI (TN)	221.14	
TSI (TP)	147.77	

TSI: Trophic State Index; TN: Total nitrogen; TP: Total phosphorus.

### 3.2 Growth alterations and toxin accumulation in lettuce cultivated with bloom-affected river water

**Fig. 1:**
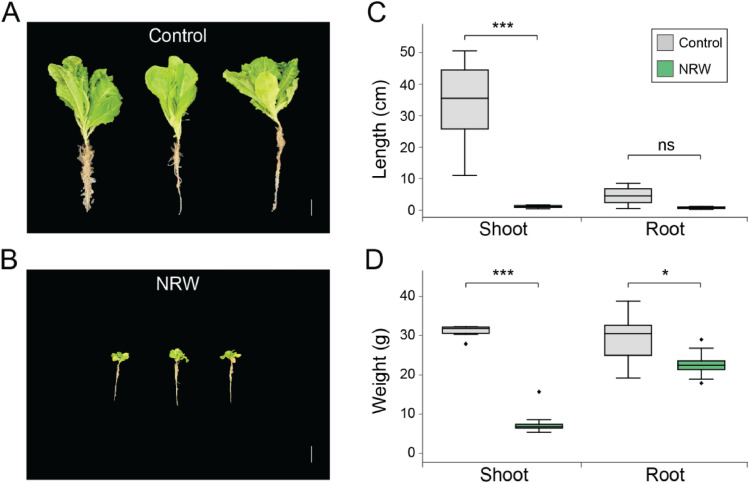
Phenotypes of lettuce in the control and NRW groups. (A, B) Representative images of lettuce plants from the control and NRW groups, respectively. Both leaf and root phenotypes are shown. The white scale bar represents 10 cm. (C, D) Box plots visualizing the quantitative data of leaf and root phenotypes. (C) Lengths and (D) weights of the leaves and roots. In both plots, gray indicates the control group, and green indicates the NRW group (n=15 plants per group from 3 biological replicates, measured at Day 30). Significant differences between groups are denoted with asterisks (* *p*< 0.05, ** *p* < 0.01, *** *p* < 0.001) based on Student’s t-test. 'ns' denotes no significant difference.

Exposure to Nakdong River water substantially altered lettuce growth patterns. Two hydroponic groups were analyzed: lettuce grown in Nakdong River water (NRW) and lettuce grown in control. Lettuce in the control group exhibited significantly greater growth than the NRW group (Fig. 1A and B). At 30 days post-planting, the mean length of the leaves in the control group was 31.05 ± 1.72 cm (Fig. 1C), whereas the NRW group displayed a markedly reduced mean leaf length of 7.18 ± 2.01 cm (*p* < 0.001, n=15 plants per group from three biological replicates). Similarly, the root lengths differed between the two groups, with a mean root length of 4.63 ± 3.09 cm in the control group compared to 0.83 ± 0.30 cm in the NRW group. Furthermore, as illustrated in Fig. 1D, the mean leaf weight in the control group was substantially higher at 33.82 ± 14.84 g in contrast to a mean leaf weight of only 1.19 ± 0.37 g in the NRW group (*p* < 0.001). The root weight also differed significantly, with the control group roots weighing 29.25 ± 7.01 g on average, while the NRW group roots weighed significantly less at 22.58 ± 2.42 g (*p* < 0.05). These findings clearly illustrate the substantial growth inhibition experienced by lettuce grown in NRW compared to those cultivated with deionized water. Additionally, the researchers evaluated the potential health hazards associated with consuming crops irrigated with cyanobacterial-contaminated water by quantifying microcystin levels in harvested lettuce leaves using LC-MS/MS analysis. In the NRW group, microcystin-LR was 5.56 μg kg^-1^ (dry wt.), and microcystin-RR was 3.99 μg kg^-1^ (dry wt.) (Table 2). Estimated daily intake (EDI) calculations, considering both MC-LR and MC-RR, showed that lettuce consumption contributed to 87.34 % (men) and 111.02 % (women) of the WHO tolerable daily intake (TDI) of 0.04 μg kg^-1^ body weight day^-1^ (Table 3).

**Table 2:** Concentrations of microcystins (MC-LR, MC-RR, and MC-YR) in the leafy parts of lettuce samples.

	Groups	
	Control	NRW
Microcystin-LR (μg kg^-1^ dry wt.)	*n.d.	5.56
Microcystin-RR (μg kg^-1^ dry wt.)	*n.d.	3.99
Microcystin-YR (μg kg^-1^ dry wt.)	*n.d.	*n.d.

Results obtained by the LC-MS/MS method; LR = leucine-arginine, RR = arginine-arginine, YR = tyrosine-arginine; *n.d. means not detected.

**Table 3:** Tolerable daily intake (TDI) and estimated daily intake (EDI) levels in cultivated lettuce samples.

	Control	NRW
WHO’s TDI (μg kg^-1^ body weight day^-1^)	-	0.04
EDI for men (74.90 kg) (μg day^-1^)	-	3.00
EDI for women (58.89 kg) (μg day^-1^)	-	2.36
Consumption of lettuce (μg day^-1^)		2.62

TDI: Tolerable Daily Intake; EDI: Estimated Daily Intake; WHO: World Health Organization; *n.d. means not detected.

### 3.3 Transcriptomic insights into alterations of energy and antioxidant metabolism by bloom-affected water

**Fig. 2:**
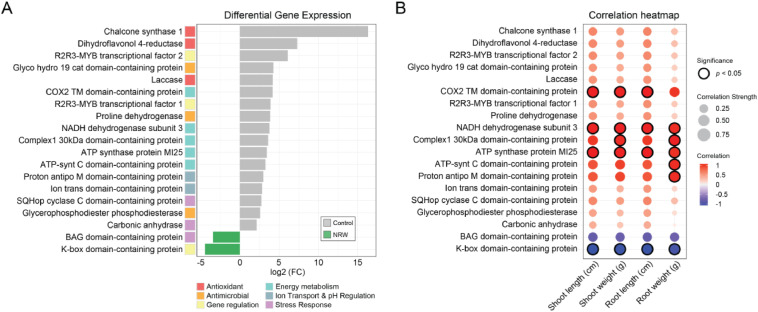
Differential gene expression and correlation with growth phenotypes in lettuce. (A) Bar graph showing log2 fold change (FC) in gene expression between the control and NRW groups. Genes are categorized by function: antioxidant (red), antimicrobial (orange), gene regulation (yellow), energy metabolism (green), ion transport & pH regulation (blue), and stress response (purple). The control group (gray bars) and NRW group (green bars) are compared. (B) Heatmap illustrating the correlation between gene expression levels and lettuce growth traits (leaf length, leaf weight, root length, root weight). Circle size indicates correlation strength, and color indicates direction (red for positive, blue for negative). Statistically significant correlations (*p* < 0.05) are outlined in black. (n=3 biological replicates per group, RNA extracted at Day 30).

Transcriptional profiling was employed to investigate the molecular responses underlying the observed growth alterations in the lettuce specimens. The distribution and statistical significance of differentially expressed genes are shown (Fig. S1A, S1B), highlighting the unique and significantly altered genes and substantial transcriptional disparities between the control and NRW groups. Analysis of gene expression revealed significant downregulation of 17 gene families across six distinct genetic pathways in the NRW group compared with the control group (Fig. 2A). Specifically, three genes associated with antioxidant function and three gene families related to antimicrobial properties were notably suppressed in the NRW group relative to the control. Five gene families involved in energy metabolism, *COX2, ND3, NDUFA9, ATP5A1*, and *ATP5C1*, encoding key components of the oxidative phosphorylation electron transport chain complexes, were significantly repressed. Additionally, gene families associated with gene regulation, ion transport, pH regulation, and stress response were predominantly expressed in the control group. Conversely, the NRW group demonstrated elevated expression of one gene related to gene regulation and another associated with the stress response compared to the control group. Notably, these changes in gene expression exhibited a robust and statistically significant correlation with the inhibited growth of lettuce leaves and roots (Fig. 2B). This strong correlation underscores the significant role these genes play in regulating growth-related processes. Taken together, these transcriptomic alterations suggest extensive impacts on the physiological processes in lettuce, thereby contributing to the observed growth inhibition.

### 3.4 Metabolomic disruptions and reduced defense compounds in lettuce

**Fig. 3:**
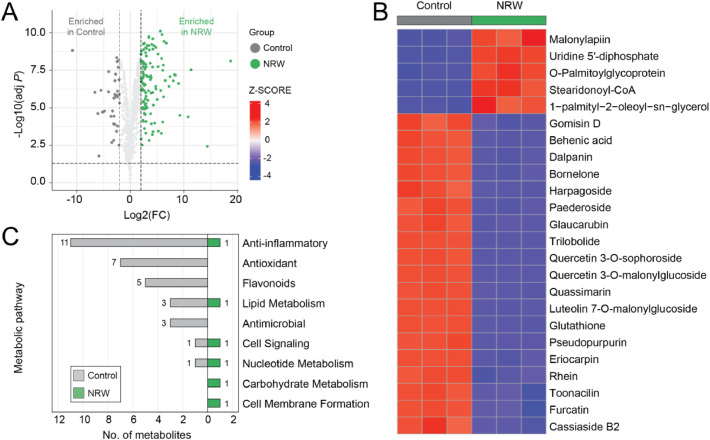
Differential metabolite expression and pathway analysis between the control and NRW groups. (A) Volcano plot showing differentially expressed metabolites between the control (gray) and NRW (green) groups, with log2(FC) on the x-axis and -log10 (adjusted P-value) on the y-axis. The horizontal dashed line represents the threshold for significance (*p* < 0.05), and the vertical dashed lines represent the log2 fold-change thresholds. (B) Heatmap of metabolite Z-scores in the control and NRW groups, with higher Z-scores in red and lower in blue. (C) Bar chart depicting the number of metabolites in different metabolic pathways for the control and NRW groups. (n=3 biological replicates per group, metabolites extracted at Day 30).

The adverse impacts of Nakdong River water contaminated with toxic cyanobacteria on plants were also evident at the metabolic level, with significant variations in metabolite profiles between the NRW and control groups due to differences in phytochemicals, as demonstrated using LC-Q-TOF mass spectrometry. Orthogonal partial least squares discriminant analysis (OPLS-DA) identified the most influential variables, focusing on metabolites with variable importance in projection (VIP) scores greater than 1 (Fig. 3A and Fig. S2). Out of 911 significantly different metabolites, 164 were cross-referenced with ChemSpider ID and KEGG pathway information to determine their relevance in lettuce metabolic pathways. A total of 24 metabolites exhibited notable differences in their abundance, which were visualized using a heatmap (Fig. 3B). The NRW group showed reduced levels of anti-inflammatory and antioxidant metabolites, including flavonoids such as quercetin 3-O-malonylglucoside, luteolin 7-O-malonylglucoside, and quercetin 3-O-sophoroside (Fig. 3C). Furthermore, antimicrobial-related metabolites were significantly diminished in the NRW group. This is consistent with transcriptomic data indicating the downregulation of flavonoid biosynthesis pathways, which suggests a compromised biochemical capacity for defense responses. Corresponding metabolites in the energy metabolism and oxidative phosphorylation pathways did not show significant changes, despite the observed downregulation of associated genes in the transcriptome analysis.

### 3.5 Shifts of microbial community in hydroponic water under cyanobacterial bloom exposure

**Fig. 4:**
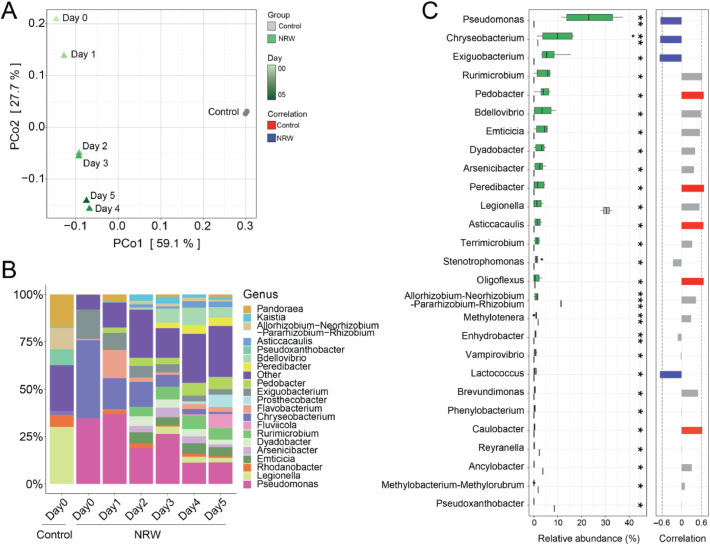
Microbial community structure in the control and NRW groups. (A) Principal coordinate analysis (PCoA) plot showing the microbial community structure. The control sample (gray) represents the Day 0 baseline, while the NRW samples (green) show a temporal trajectory from Day 0 to Day 5. The separation along the PCo1 and PCo2 axes explains 59.1 % and 27.7 % of the variation, respectively. (B) Stacked bar plot illustrating the relative abundance of bacterial genera in the control and NRW groups across different days (Day 0 to Day 5). Each color represents a different genus. (C) Bar plot showing the relative abundance of significantly different genera between the control and NRW groups. Genera with significant differences are marked with asterisks (* *p*< 0.05, ** *p* < 0.01, *** *p* < 0.001). The right panel indicates the correlation of these genera with experimental conditions, with red and blue bars representing positive and negative correlations, respectively. (NRW group: n=3 biological replicates per day from Day 0-5; Control group: n=3 biological replicates at Day 0).

Given the observed abundance of antimicrobial-related genes and metabolites in lettuce, we investigated the microbiome of the water in the hydroponic systems. 16S rRNA amplicon sequencing was performed daily over a period of 5 days to analyze the bacterial communities in both the NRW and control groups. Microbiome analysis based on Bray-Curtis dissimilarity revealed clear separation between the microbial communities of the NRW and control groups (PERMANOVA, *p* < 0.01) (Fig. 4A), with slightly higher microbial diversity in the NRW group (Fig. S3A) PERMANOVA analysis revealed significant differences in overall microbial community structure between NRW and control treatments. Additionally, the PCoA plot shows a clear temporal trajectory within the NRW group from Day 0 to Day 5, indicating dynamic community changes over time. These differences were evident at both the phylum and genus levels of microbial classification (Fig. 4B and Fig. S3B). A striking observation was the dramatic decline of cyanobacteria from the source river water to the hydroponic system. While the initial Nakdong River water contained high densities of cyanobacterial cells (144,062 cells/mL, Table 1), predominantly comprising Microcystis and Dolichospermum, these photoautotrophic organisms failed to establish substantial populations within the hydroponic environment, declining to approximately 1-2 % at the phylum level (Fig. S3B) and becoming negligible at the genus level (grouped in 'Other', Fig. 4B). This rapid decline represents a fundamental ecological shift indicative of microbial dysbiosis. The NRW group had a higher relative abundance of bacterial genera such as *Pseudomonas, Chryseobacterium*, and *Exiguobacterium,* whereas the control group had a higher abundance of *Pseudoxanthobacter, Methylobacterium-Methylorubrum*, and *Ancylobacter* (Fig. 4B and C). Pseudomonas, Chryseobacterium, and Exiguobacterium were prevalent in the NRW group but showed a decreasing trend over time, indicating a negative correlation with the duration of exposure. Conversely, Pedobacter, Peredibacter, and Asticcacaulis in the NRW group exhibited an increasing trend over time, indicating a positive correlation with the duration of exposure. No notable changes in these genera were observed in the control group.

## 4 Discussion

Although the adverse effects of cyanotoxins and cyanobacterial blooms on crop development have been reported (Abo-Shady et al., 2023; Bakr et al., 2022; Campos et al., 2021), the specific ways through which these blooms affect plant growth remain complex and not fully understood. To address this knowledge gap, growth alterations in lettuce cultivated with bloom-affected Nakdong River water were examined using a controlled hydroponic system allowing for precise assessment of plant responses while minimizing soil-related confounding variables. This approach allowed for in-depth analyses of gene expression, metabolite profiles, and microbial community changes, leading to the characterization of interconnected physiological and molecular responses contributing to the observed phytotoxicity.

To simulate real-world agricultural exposure scenarios, the collected Nakdong River water samples, characterized by high levels of nutrient pollution and microcystin contamination, were considered appropriate for use in hydroponic cultivation experiments. TSI (Carlson, 1977; Cunha et al., 2013) calculations further confirmed the severe eutrophic state of the water. The TSI for total phosphorus was 148, while the TSI for total nitrogen was 221, both of which are significantly higher than the commonly recognized thresholds of 50-70 for hyper-eutrophic conditions (Lin et al., 2022). These elevated TSI values far exceed those reported in other eutrophic water bodies, such as Lake Bunyonyi in Uganda, where the TSI-TP ranges from 58 to 65 (Saturday et al., 2023), or drinking water reservoirs in Taiwan, where the TSI-TP is typically below 100 (Lin et al., 2022). The nutrient levels in the Nakdong River are thus substantially greater, leading to an environment highly conducive to harmful algal blooms and subsequent phytotoxic effects (Lee et al., 2018). This severe eutrophication not only exacerbates the proliferation of cyanobacteria but also establishes a direct pathway for transferring these complex stressors into agricultural production, raising dual concerns: the public health risk from toxin accumulation in edible crops and the largely uncharacterized physiological impact on the plants themselves, which forms the central inquiry of this study. Therefore, the relationship between the application of bloom-affected water and the observed phytotoxicity is indirect, reflecting the net impact of a complex mixture of stressors and nutrients.

These findings suggest severe ecological disruption and significant public health concerns. Consistent with previous studies, this study confirmed the uptake and translocation of microcystins into the leaf part (edible part) of the harvest lettuce. The daily intake of microcystin from lettuce was estimated to be 2.62 μg day^-1^ for Korean adults aged 20 to 60 years, based on the WHO TDI guidelines (Table 3) (WHO, 2020). In the present study, the EDI of microcystins was approximately 3.00 μg day^-1^ for men and 2.36 μg day^-1^ for women, with the experimental lettuce intake constituting 87 % of the EDI for men and exceeding it for women. Unlike previous studies using purified toxins or isolated cyanobacterial species, this study demonstrates that crops cultivated with naturally bloom-affected river water can accumulate various compounds including microcystins. The fact that this accumulation reaches levels of public health concern provides a critical real-world context for our central investigation: understanding the comprehensive biological disruptions within the plant that lead to such outcomes.

Specifically, this study focused on investigating the comprehensive effects of bloom-affected water, which contains complex mixtures of nutrients, toxins, and other bioactive compounds during bloom events, on the development of a leafy crop. Microcystins are predominantly known for their deleterious effects on chloroplasts (Máthé et al., 2021; Welker et al., 2003), where they disrupt photosynthetic efficiency and elevate levels of reactive oxygen species (ROS), leading to significant oxidative stress and subsequent cellular damage (Bittencourt-Oliveira et al., 2014). However, this study identified a critical, and somewhat underappreciated, aspect of bloom water effects: the profound impact on mitochondrial function (Nugumanova et al., 2023). A substantial downregulation was observed in key mitochondrial genes involved in oxidative phosphorylation (*COX2, ND3, NDUFA9, ATP5A1*, and *ATP5C1*) (Máthé et al., 2021). This pattern suggests that bloom water components not only compromise chloroplast function but also significantly impair mitochondrial ATP production (Máthé et al., 2021).

The impairment of mitochondrial function observed in plants exposed to bloom-affected water is concerning as it disrupts the plant's delicate energy balance (He et al., 2023; Sun et al., 2021). Chloroplasts and mitochondria are interdependent (Snyder and Stefano, 2015); chloroplasts generate substrates for ATP production, while mitochondria provide the ATP required for critical chloroplast functions, including the Calvin cycle (Snyder and Stefano, 2015). Downregulation of mitochondrial genes compromises energy production, thereby microcystin-induced damage. These findings emphasize the need to consider the interdependent roles of chloroplasts and mitochondria in maintaining cellular energy balance when plants are exposed to complex bloom-affected water. Interestingly, despite the pronounced downregulation of these mitochondrial genes, the corresponding metabolites in the energy metabolism and oxidative phosphorylation pathways did not exhibit immediate changes. This apparent discrepancy might reflect a temporal delay, in which transcriptional changes precede detectable shifts in metabolite pools. Alternatively, it could indicate the activation of short-term compensatory mechanisms at the post-transcriptional or translational level to maintain metabolic homeostasis against the initial shock, highlighting the potential resilience of plants to transient disturbances. Future longitudinal studies are needed to distinguish between these possibilities and provide deeper insights into these temporal dynamics.

Beyond the disruptions in energy metabolism, the study uncovers another critical aspect of bloom water effects: the direct compromise of the plant's antioxidant defenses. While the impairment of mitochondrial function primarily affects energy production, the simultaneous downregulation of key antioxidant genes exacerbates the vulnerability of the plant to oxidative damage (Adamski and Kaminski, 2022; Juhász et al., 2023). This dual impact creates a scenario in which the plant is not only struggling with energy deficits but is also increasingly unable to defend itself against the ROS generated under stress conditions (Rajput et al., 2021). Specifically, the findings show a substantial reduction in the expression of superoxide dismutase (SOD) and catalase (CAT), enzymes that are vital for detoxifying ROS (Kerchev and Van Breusegem, 2022). The downregulation of these genes indicates a disruption in the plant’s primary oxidative stress defense mechanisms, leading to elevated ROS accumulation (Jiang et al., 2022; Zhang et al., 2022). Concurrently, ROS enrichment can reshape microbiome in the hydroponic solution toward a dysbiotic state, selectively favoring facultative or opportunistic taxa that thrive under oxidative stress (Berrios and Rentsch, 2022). This feedback loop links antioxidant depletion to plant–microbe dysbiosis in the water column, thereby magnifying toxin-induced damage. Therefore, the plant is caught in a vicious cycle where metabolic stress and microbial imbalance reinforce each other.

What distinguishes this study is the observation that this antioxidant system impairment occurs concurrently with mitochondrial dysfunction, resulting in a compounded stress response. Unlike studies that have focused on single pathways, this research highlights the interconnected nature of these systems. The impaired antioxidant defenses mean that the plant cannot effectively neutralize the ROS generated by compromised chloroplast and mitochondrial activities, leading to a feedback loop of escalating damage. Furthermore, the reduction in antioxidant metabolites, particularly flavonoids, underscores the extent of this disruption (Sachdev et al., 2021). Flavonoids not only scavenge ROS but also support other antioxidant enzymes in maintaining the cellular redox balance (Li et al., 2023). Their depletion implies a broader destabilization of the antioxidant defense network, which is critical for sustaining plant resilience under environmental stress.

This dual impairment of mitochondria and antioxidant defenses provides a more comprehensive understanding of how bloom-affected water impacts plant systems (Campos, 2021). These findings underscore the need for integrated strategies that enhance both energy production and antioxidant capacity to confer resistance against microcystin-induced stress. Addressing both pathways may offer a more robust means of protecting crops from the multifaceted threats posed by bloom-affected water.

In addition, the results also demonstrate that bloom water alters beneficial communities of microbes, which are essential for plant growth and health. These beneficial microbes are known to assist with nutrient uptake, stress tolerance, and disease resistance (Kumar and Dubey, 2020; Poppeliers et al., 2023). However, exposure to bloom-affected water induced a shift in structure and composition of microbial community. This shift was characterized by a decline in beneficial symbiotic taxa and an enrichment of harmful genera, representing not just a taxonomic change but a potential collapse of the functional network supporting the plant. These shifts collectively exacerbated stress conditions and impaired plant development. This reconfiguration represents a plant–microbe dysbiosis, in which the mutualistic network between lettuce and its rhizosphere partners collapses, amplifying physiological stress (Darriaut et al., 2024).

Within this dysbiotic profile, an increased abundance of stress-tolerant bacterial genera such as *Pseudomonas* and *Chryseobacterium* (Islam and Sandhi, 2023; Koza et al., 2022) was observed in association with bloom-affected treatments. These genera are well-adapted to thrive in challenging conditions where other beneficial microbes struggle. However, their proliferation in the rhizosphere can harm plant health (Costa-Gutierrez et al., 2022; Singh et al., 2023). For example, *Pseudomonas* species, though sometimes beneficial, can become opportunistic pathogens that produce toxins or aggressively compete for limited nutrients, thereby with plant growth and exacerbating stress (Chepsergon and Moleleki, 2023). Conversely, the abundance of beneficial microbes such as *Pedobacter* and *Asticcacauli* (Chepsergon and Moleleki, 2023; Herpell et al., 2023; Wang et al., 2021), which are known for their roles in nutrient cycling and promoting plant growth, was significantly reduced (Singh et al., 2023), implying a diminished capacity for key supportive functions like phosphate solubilization and phytohormone production. The disruption of the microbial community in the hydroponic system highlights an underappreciated axis of plant stress physiology. The observed shift toward stress-tolerant or potentially harmful microbes in response to microcystin exposure paints a fascinating picture. It is as if the toxins not only directly undermine the plant's defenses, but also reshape the very ecological environment that the plant depends on (Bartas, 2024). This cascading effect, where the stressed plant favors the growth of less beneficial microbes, which in turn further compromise the plant's well-being, creates a captivating cycle of decline (Ali et al., 2025). It is a sobering reminder that the impact of environmental toxins can reverberate far beyond the plant itself, rippling through the intricate web of ecological relationships. This microbial dimension introduces a previously underappreciated axis of plant stress physiology, potentially compounding the direct effects of microcystins (Núñez et al., 2024).

Understanding plant-microbe-environment interactions is critical for fully assessing the risks posed by bloom-affected water (Li et al., 2022; Suman et al., 2022). A holistic perspective will be essential for developing mitigation strategies, including targeted microbial inoculants and cultivation practices that foster beneficial microbiomes. The current findings emphasize that the effects of cyanobacterial blooms are not limited to internal physiological disruption but extend to ecological imbalances in the plant's microbiome. Addressing these microbial shifts is thus pivotal for protecting crop productivity in contaminated systems.

While this study provides valuable insights, several limitations should be noted. First, the relatively small sample size may constrain the generalizability of the results. Second, our analysis was limited to lettuce leaf tissues, and root tissues were not analyzed, despite their direct contact with contaminated water and critical role in nutrient uptake and microbial interactions. Our microbiome analysis was limited to the hydroponic water to characterize the primary environmental stressor, and a comprehensive analysis of the root- and leaf-associated microbiomes is a critical next step. Similarly, while our metabolomic analysis was focused on phytochemicals, we cannot entirely exclude minor contributions from the phyllosphere microbiome to the overall metabolite pool. Third, the control and NRW treatments differed in both toxin content and nutrient composition, making it difficult to isolate the specific effects of cyanotoxins from nutritional differences. However, this experimental design reflects realistic agricultural conditions where bloom-affected water sources present complex mixtures of both beneficial nutrients and harmful compounds. It is also important to acknowledge that our study did not measure other potential co-stressors in the river water, such as heavy metals, pesticides, or other organic pollutants, which could have acted synergistically to impact lettuce growth. Fourth, our study used water from a single collection event stored at 4 °C in the dark for up to 35 days. While this ensured reproducibility, storage conditions may have altered microbial community structure and cyanobacterial viability compared to using fresh river water directly. Our results therefore represent the effects of stored bloom-affected water, which may differ from continuous irrigation with fresh water in field settings. The observed effects represent the net impact of using such water sources, which is the practical concern for agricultural decision-making. Future research should include comprehensive root-specific analyses and controlled studies using purified toxins to achieve a more complete understanding of cyanotoxin-specific effects, while our approach offers valuable insights into the real-world challenges faced by farmers who are choosing between expensive, nutrient-balanced solutions and readily available natural water sources during bloom seasons.

This study provides a novel systems-level perspective into how bloom-affected water disrupts plant function, suggesting that direct physiological stress on the plant occurs concurrently with a significant disruption of the surrounding aquatic microbial community. In addition, this study makes several important contributions to the agricultural field. By integrating transcriptomic, metabolomic, and microbiome analyses, it provides a comprehensive overview of how compounds from cyanobacterial blooms affect plant health at multiple biological levels. The findings highlight the synergistic effects of mitochondrial dysfunction, compromised antioxidant responses, and shifts in microbial communities, all of which exacerbate plant stress and contribute to growth alterations. Moreover, the precise control provided by the hydroponic system enabled clear interpretation of the results by minimizing extraneous environmental variables. The toxin extraction and analytical methods were also carefully validated and followed the standard protocols widely accepted in existing literature, ensuring high reliability and reproducibility of toxin quantification data.

Understanding this tripartite threat (impaired energy, weakened defenses, and microbial dysbiosis) can inform the development of targeted, multi-pronged mitigation strategies. For instance, interventions could focus on applying biostimulants to support energy metabolism, using exogenous antioxidants to bolster plant defenses, or developing probiotic inoculants to restore a healthy microbial community (Khoulati et al., 2025; Nephali et al., 2021). Such knowledge is crucial for ensuring sustainable agricultural practices, and the insights gained from this research offer a foundation for future studies aimed at improving crop resilience in contaminated environments.

In summary, our data support a comprehensive effects model whereby bloom-affected water exposure influences multiple biological pathways, including energy metabolism, antioxidant systems, and plant-associated microbial communities, collectively impacting lettuce growth and physiological responses. Our findings establish a strong correlation between irrigation with bloom-affected water and compromised plant health, rather than a direct causal link to cyanotoxins alone. This framework predicts field-level risks and guides the design of microbiome-based corrective strategies.

## Funding

This research was supported by Basic Science Research Program through the 10.13039/501100003725National Research Foundation of Korea (NRF) grant funded by Korea government (MSIT) (No. 2022R1C1C10087991361782064340103).

Data Availability: Raw sequencing data from the lettuce root RNA-seq and aquatic microbiome in hydroponic system experiments are available in the NCBI Sequence Read Archive (SRA) under the BioProject accessions PRJNA1269643 and PRJNA1269763, respectively.

## Author contribution statement

**Minsoo Jeong**: Writing – original draft, Formal analysis

**Soeun Park**: Data curation, Formal analysis

**Seungjin Jeong**: Formal analysis, Writing - review & editing

**Surye Park**: Formal analysis and data curation

**Sohyun Yeo**: Data curation

**Bomi Ryu**: Writing - review & editing

**Jae-Ho Shin**: Supervision, Funding acquisition, Writing - review & editing

**Seungjun Lee**: Supervision, Conceptualization, Writing - review & editing, Funding acquisition

## Declaration of competing interest

The authors declare that they have no known competing financial interests or personal relationships that could have appeared to influence the work reported in this paper.

## Appendix Supplementary materials

Image, application 1
