# Supplementary material for: Multi-omics insights into plant-microbe dysbiosis caused by cyanobacterial bloom-affected water
Source: Curr Res Microb Sci. 2025 Oct 23;9:100500. doi: 10.1016/j.crmicr.2025.100500 (PMC12615344; doi:10.1016/j.crmicr.2025.100500)
Supplement: Supplementary file 1 [file mmc1.docx]

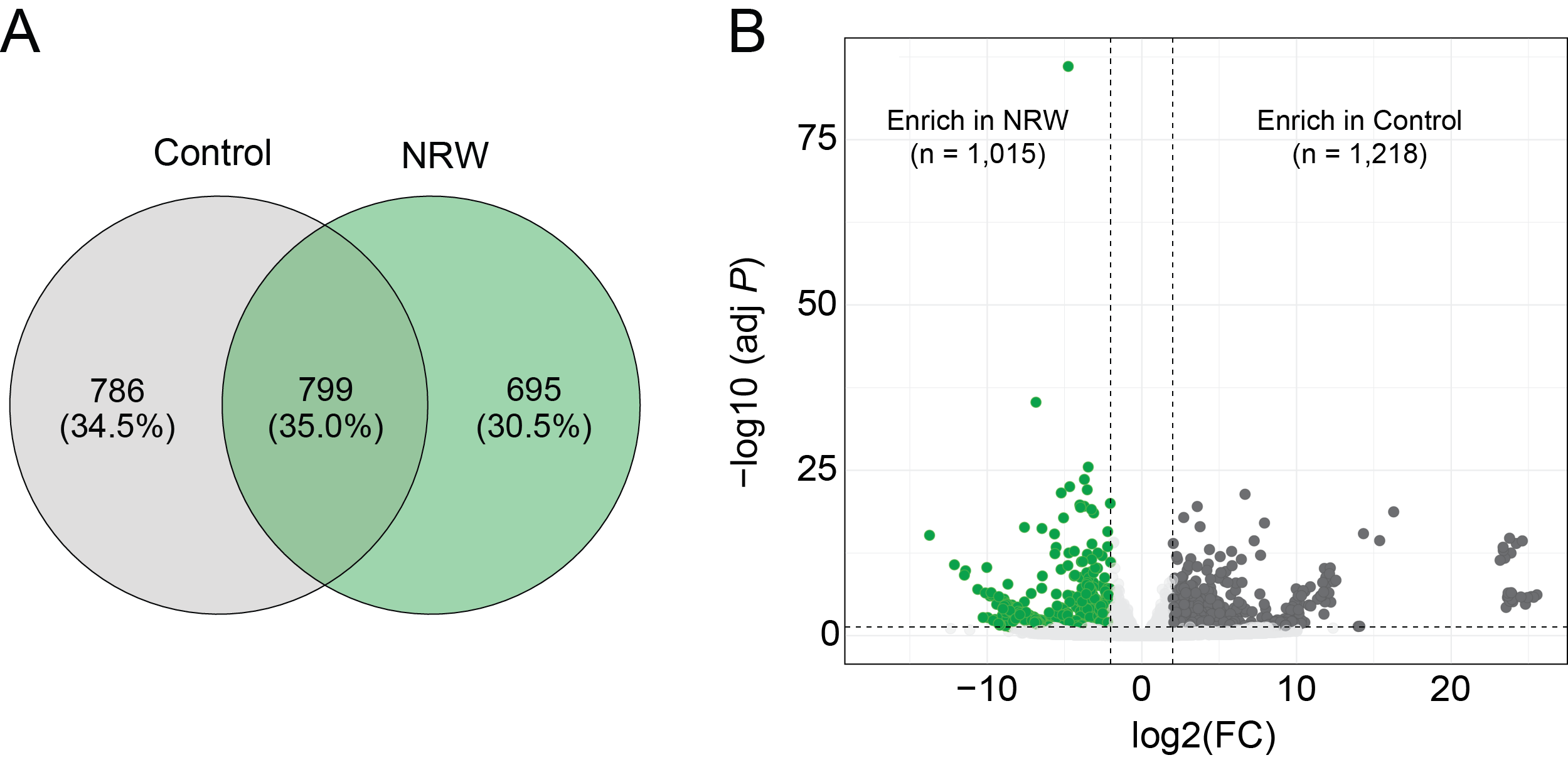


**Fig. S1**. Differentially expressed genes in the control and NRW groups. (A) Venn diagram showing the distribution of differentially expressed genes between the control and NRW groups. (B) Volcano plot illustrating the log2 fold change (FC) versus -log10 adjusted *p*-value for genes in the control and NRW groups. Genes enriched in the NRW group (green) and control group (gray) are indicated, with vertical dashed lines representing the thresholds for significant differential expression.


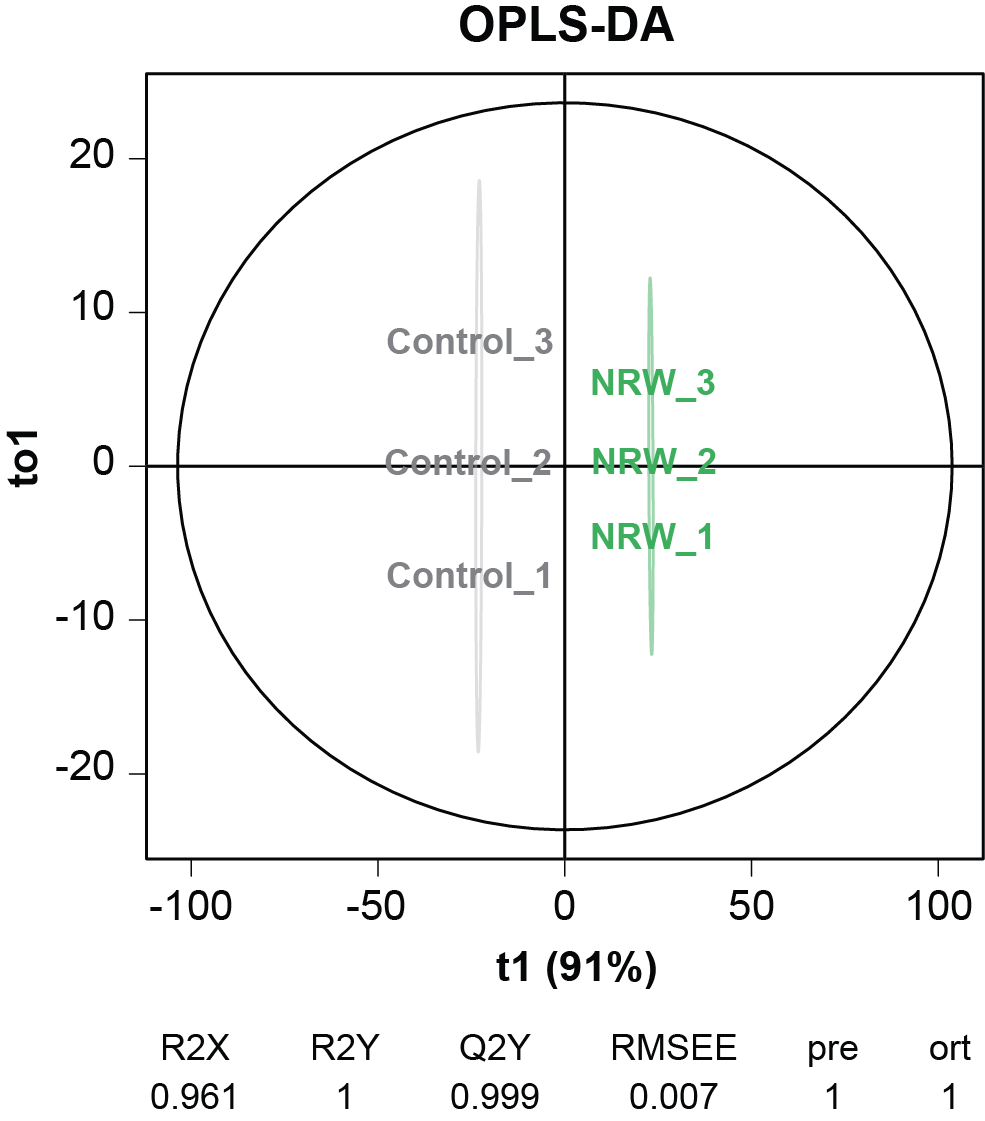


**Fig. S2. Orthogonal partial least squares discriminant analysis (OPLS-DA) score plot. This plot shows the separation between the control (gray) and** NRW **(green) sample groups based on their metabolic profiles. The control group includes samples control_1, control_2, and control_3, while the** NRW **group includes samples** NRW **_1,** NRW**_2, and** NRW**_3. The horizontal axis (t1) accounts for 91% of the variance, and the vertical axis (to1) captures the orthogonal component. Key statistics:** R2X = 0.961 (explained variance in the predictors), R2Y = 1.000 (explained variance in the response), Q2Y = 0.999 (predictive ability), RMSEE = 0.007 (root mean square error of estimation)**, predictive value = 1, and orthogonality = 1.**


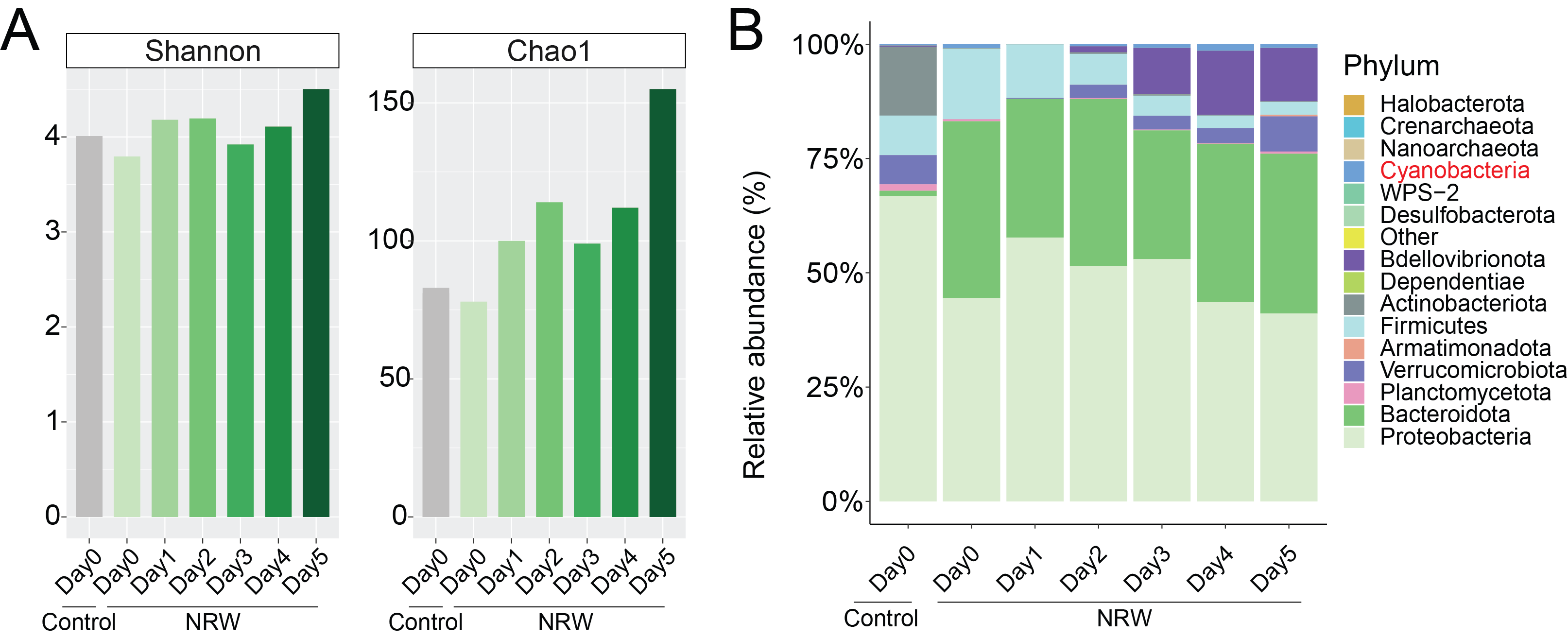


**Fig. S3**. Microbial community structure and diversity in the control and NRW groups. (A) Alpha diversity: bar plots showing Shannon and Chao1 diversity indices for the control and NRW groups across different days (Day 0 to Day 5). (B) Stacked bar plot showing the relative abundance of microbial phyla in the control and NRW groups across different days (Day 0 to Day 5). Each color represents a different phylum. The Cyanobacteria phylum is highlighted in red to emphasize its dramatic decline from high abundance in the source river water (144,062 cells/mL, Table 1) to minimal presence (<2%) in the hydroponic system.

**Table S1**. Elemental composition of the hydroponic nutrient solution stock (A and B, Daeyu Co., South Korea) used for the control group.

The commercial hydroponic fertilizer used in this study is formulated based on the standard Hoagland solution. The nutrient solution was prepared by diluting Stock A and Stock B according to the manufacturer's instructions to achieve final concentrations optimal for lettuce cultivation.

| Nutrient Element | Stock A  Concentration (mg/L) | Stock B  Concentration (mg/L) | Final Working  Concentration (mg/L) | Chemical Form |
| --- | --- | --- | --- | --- |
| Nitrogen (N) | 210 | - | 210 | NH₄NO₃, Ca(NO₃)₂ |
| Phosphorus (P) | 31 | - | 31 | NH₄H₂PO₄ |
| Potassium (K) | 234 | - | 234 | KNO₃, K₂SO₄ |
| Calcium (Ca) | - | 200 | 200 | Ca(NO₃)₂·4H₂O |
| Magnesium (Mg) | - | 48 | 48 | MgSO₄·7H₂O |
| Sulfur (S) | 64 | - | 64 | K₂SO₄, MgSO₄ |
| Iron (Fe) | 2.8 | - | 2.8 | Fe-EDTA |
| Manganese (Mn) | 0.55 | - | 0.55 | MnSO₄·H₂O |
| Zinc (Zn) | 0.26 | - | 0.26 | ZnSO₄·7H₂O |
| Copper (Cu) | 0.08 | - | 0.08 | CuSO₄·5H₂O |
| Boron (B) | 0.5 | - | 0.5 | H₃BO₃ |
| Molybdenum (Mo) | 0.05 | - | 0.05 | Na₂MoO₄·2H₂O |

**Note**: Stock A and Stock B are kept separate to prevent precipitation of calcium and phosphate. The working solution was prepared by combining appropriate volumes of Stock A and Stock B with deionized water according to the manufacturer's protocol, achieving a final electrical conductivity (EC) of approximately 1.5-2.0 dS/m and pH of 5.8-6.2, which are optimal ranges for hydroponic lettuce cultivation.
